# An Update on Non-Invasive Skin Imaging Techniques in Actinic Keratosis—A Narrative Review

**DOI:** 10.3390/medicina60071043

**Published:** 2024-06-26

**Authors:** Katarzyna Korecka, Dominika Kwiatkowska, Ewelina Mazur, Aleksandra Dańczak-Pazdrowska, Adam Reich, Ryszard Żaba, Adriana Polańska

**Affiliations:** 1 Department of Dermatology, Poznań University of Medical Sciences, 61-545 Poznań, Poland; aleksandra.danczak-pazdrowska@ump.edu.pl (A.D.-P.); apolanska@ump.edu.pl (A.P.); 2 Department of Dermatology, Institute of Medical Sciences, Medical College of Rzeszow University, 35-959 Rzeszow, Poland; 3 Doctoral School, University of Rzeszow, 35-310 Rzeszow, Poland

**Keywords:** actinic keratosis, noninvasive skin imaging, dermoscopy, high-frequency ultrasonography, reflectance confocal microscopy

## Abstract

Nonmelanocytic skin cancers (NMSCs) are currently the most common group of human cancers and include all tumors that are not melanomas. Increased exposure to sunlight over the past few years, the lack of regular and proper use of sunscreen, the aging of the population, and better screening techniques are the reasons for the escalation in their diagnosis. Squamous cell carcinoma (SCC) comprises nearly 37% of the tumors in this group and can originate from actinic keratosis (AK), which usually presents as pink, often scaly plaques, usually located on the face or scalp. Advances in dermatoscopy, as well as the development of other non-invasive skin imaging modalities such as high-frequency ultrasound (HFUS), reflectance confocal microscopy (RCM), and optical coherence tomography (OCT), have allowed for greatly increased sensitivity in diagnosing these lesions and monitoring their treatment. Since AK therapy is usually local, and SCCs must be removed surgically, non-invasive imaging methods enable to correctly qualify difficult lesions. This is especially important given that they are very often located on the face, and achieving an appropriate cosmetic result after treatments in this area is very important for the patients. In this review, the authors describe the use of non-invasive skin imaging methods in the diagnosis of actinic keratosis.

## 1. Introduction

### 1.1. Introduction to NMSC

Nonmelanocytic skin cancers (NMSCs) are currently the most common group of human cancers and include all tumors that are not melanomas. Since most of them occur in Caucasian patients, they are also called “white skin cancer” [1]. The increase in their diagnosis is associated with a longer life expectancy in the population, and they have a genetic background or are related to immunosuppression. However, the most significant cause of their occurrence is ultraviolet (UV) exposure [2]. The most common neoplasm in this group is basal cell carcinoma (BCC), which comprises about 63% of these tumors [3].

Squamous cell carcinomas (SCC) represent about 37% of diagnosed NMSC and can arise in areas of actinic keratosis (AK) or de novo [3]. Depending on a study, 60–80% of SCCs might develop on the basis of AK [4].

### 1.2. Demographics and Clinical Presentation of AKs

Clinically, AKs usually present as erythematous plaques located on the face, scalp, and palmar surface of the hands, neck, or lips. They are usually found in elderly patients with a fair skin phototype and visible photodamage. The incidence of AK varies and depends on a geographical location—in Australia, it is present in 40–60% of the white inhabitants, while in Europe, AK might be seen in 4.7% up to 37% of the population [5]. Their differential diagnosis is wide and includes SCC in situ (Bowen’s disease), superficial BCC, and seborrheic keratosis [6]. AKs may regress spontaneously (20–30% of single lesions) [5], remain on the skin, and progress to SCC in situ or an invasive tumor in 0.1–20% of the cases [7,8,9]. The rate of the progression of a single lesion to a malignant tumor was estimated at about 0–0.53% per year [10]. However, a higher number of lesions (>10) increases a patient’s cumulative risk of malignancy by up to 14% over the next 5 years. [7]. 

Some patients present with multiple AKs, most often on the scalp, and this phenomenon is known as “field cancerization”. Its concept corresponds to the fact that due to extensive UV exposure, the cutaneous tissue develops fields of cells predisposed to malignant transformation. Clinically, actinic damage might be seen. Since the entire skin is affected by ultraviolet radiation, subclinical atypia surrounds the visible AKs and possibly precedes their development [11]. 

### 1.3. Pathogenesis of AK

The main factor for developing actinic keratoses is UV radiation. After the intermittent exposure to sunlight and the constant oxidative stress that occurs in the cells, the dysplastic intraepidermal keratinocytes proliferate, and this process is enhanced by factors such as DNA damage, inflammation, immunosuppression, and mutagenesis [12]. This phenomenon also occurs on the molecular level since UVB can affect the tumor suppressor proteins such as p53, p16INK4a, and PTEN, which also contribute to the progression to a malignant entity [13]. 

A factor that increases the risk of developing AKs and their transformation to SCC is immunosuppression, so transplant patients are a special group requiring observation, as well as patients with acquired autoimmune deficiency, leukemia, and those who are treated with chemotherapy or other drugs interfering with the immune system (especially in the era of biologicals) [14,15,16]. 

### 1.4. Classification and Grading of AK

Typically, AKs are diagnosed clinically and are graded according to a classification introduced by Olsen et al., which is based on an assessment of the thickness of the lesion and the presence of scales [17]. Lesions classified as grade 1 are invisible but palpable; in grade 2, they are visible and palpable, while in grade 3, they are very thick and hyperkeratotic [17]. The main limitation of this method is that it only evaluates AKs according to their total thickness and focuses on a single lesion. However, it does not consider the extent of the involved area. To address this problem, new scales, the so-called Actinic Keratosis Field Assessment Scale (AK-FAS) [18] and Actinic Keratosis Area and Severity Index (AKASI) were created to quantify the involvement of the face and scalp [19]. In AK-FAS, the total skin area affected by AK lesions, hyperkeratosis, and sun damage are examined. To assess AKASI (Table 1), the scalp is divided into four sections, and each section is assessed for the approximate severity of the three characteristic clinical symptoms of AK (distribution, erythema, and thickness). The study by Schmitz et al. indicates that AKASI can be helpful in assessing the risk of AK transformation to a malignant tumor. Patients with a score higher than 7 were more likely to develop SCC, suggesting that this group requires special attention and regular monitoring [20].

Recently, the Method of Assessing Skin Cancerization and Keratoses (MASCK) was also developed [21]. It was created to provide a comprehensive score that objectively assesses the severity of extensive skin field cancerization area on any anatomical site or zone pre or post-treatment. It’s a promising tool in clinical practice that takes into consideration the examination of cancer in the zone, a factor absent in AKASI or AK-FAS, and is also the first to focus on any anatomical sites.

For many years, lesions graded as AK I were considered ‘low risk’. However, in a study by Fernandez-Figueras et al. [22], most of the SCCs developed on AK background consisted of overlaying AK I in the biopsy, which suggests that all AK lesions have the potential to transform into a malignant tumor. A “differentiated pathway” model was proposed and focuses on the fact that atypical keratinocytes migrate downwards along hair follicles and sweat ducts. The abnormal cells are located deep and might be resistant to treatment. This mechanism was most commonly seen in AK I [22].

The histopathological classification of AK is called Keratinocyte Intraepithelial Neoplasia (KIN). It is divided into three categories, which stratify the degrees of epidermal dysplasia. In KIN I, the atypical keratinocytes are confined to the lower third of the epidermis, while in KIN II, they are confined to the lower two-thirds. In KIN III, including Bowen’s disease, cellular atypia occurs throughout the thickness of the epidermis, while no infiltration of atypical cells into the dermis is observed [23]. Subsequently, the clinical classification does not match the histopathological grade of the lesion [24]; thus, it is important to treat every AK lesion as well as field cancerization. 

### 1.5. Treatment of AK

Therapy for AKs starts with sun avoidance and the use of sunscreens, which slows the appearance of AKs in patients who are already affected by them [25]. The treatment modalities for AK are usually non-invasive, so proper classification of the lesions is particularly important. 5-fluorouracil, imiquimod, 5% diclofenac sodium in 2.5% hyaluronic acid, and chemical peels might be used. Moreover, cryosurgery and electrocoagulation are also widely applied. Photodynamic therapy, as well as 5-fluorouracil, imiquimod, and 5% diclofenac sodium in 2.5% hyaluronic acid, have good efficacy in treating field cancerization [26]

Since AK is one of the most common reasons for dermatological consultations, it is extremely important to diagnose them efficiently and quickly. The main purpose of prompt treatment is to prevent tumor formation. Although histologic examination remains the gold standard for diagnosis, non-invasive imaging techniques are a valuable and helpful asset in making the correct diagnosis and, in doubtful cases, in selecting the appropriate site for a biopsy.

## 2. Actinic Keratosis in Dermoscopy

Dermatoscopy is an auxiliary diagnostic technique based on the Tyndall effect and Rayleigh scattering phenomenon. It has been continuously developing over the past few years and enables to examine structures invisible to the naked eye. Nowadays, its application includes not only cancers but also inflammatory and infectious diseases. It is a quick and widely available method that allows efficient and non-invasive evaluation of all, not only suspicious, skin lesions [27,28]. Dermatoscopy allows visualization of skin structures with polarized and non-polarized light at 6- to 100-fold magnification, reaching down to the papillary layer of the dermis [27]. It has been proven to increase the sensitivity and specificity of diagnosing melanomas and NMSCs [28,29,30,31,32] and facilitates the selection of skin lesions for excisional biopsy or topical treatment with cytostatic agents.

**Figure 1:**
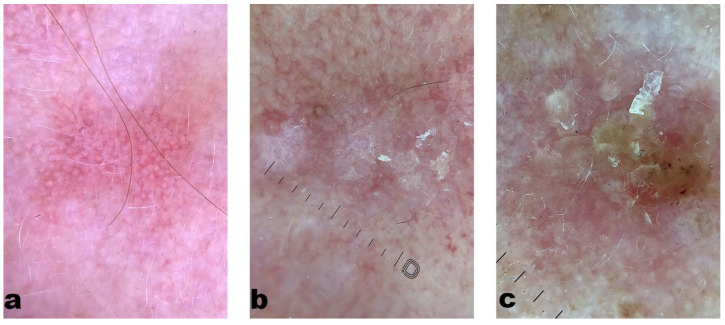
Dermatoscopic image of actinic keratosis: (magnification 10×) (**a**) Grade 1: a pattern of pink or red pseudonetwork with discrete white scales. (**b**) Grade two has an erythematous background alongside white or yellow, keratinized, and wide hair follicles (commonly known as the “strawberry pattern”). (**c**) Grade 3: wide hair follicles filled with keratinized masses on a scaly, white-yellow background.

Zalaudek’s [9] clinical-dermatoscopic classification of AK is associated with different stages of its progression. In grade one (Figure 1a), a pattern of pink or red pseudonetwork with discrete white scales is usually noticeable. Grade two (Figure 1b) is characterized by an erythematous background alongside white or yellow, keratinized, and wide hair follicles (commonly known as the “strawberry pattern”). In grade three (Figure 1c), wide hair follicles filled with keratinized masses on a scaly, white-yellow background are described (Figure 1) [33]. In this stage, hyperkeratosis can also be observed, usually as whitish, yellow, and structureless areas (Table 1). Other typical features of AK are four dots, visible only under polarized light, commonly referred to as “rosettes”, shiny streaks, and linear, wavy vessels surrounding the hair follicles. In the pigmented variant of AK, brown pseudonetwork and granular-annular homogeneous areas can be observed [4]. Giacomel et al. [34] suggested that in patients with multiple AKs, the lesions usually follow a similar dermatoscopic pattern—patients with fair skin phototypes tend to develop non-pigmented lesions, while those with higher phototypes usually present with multiple pigmented AKs.

Since distinguishing AK from SCC in situ is sometimes difficult, some literature reports have described dermatoscopic features to differentiate between these two entities. Zalaudek et al. [35] proposed a progression mode of facial AK developing into SCC in situ and then the invasive form. The AKs that displayed atypia usually presented with vessels around follicles that became dotted, and then, as the lesion evolved, they became more convoluted, glomerular, and clustered [35]. The main markers of early SCC in situ lesions reported by Papageorgiou et al. were white, structureless areas, with 70% of cases showing dotted vessels and 40% displaying curved vessels [36]. Another noticeable feature described was the production of a keratin mass, which is distributed in the central part of the lesion. In an invasive tumor, ulceration may appear. However, this is not a typical SCC feature and may occur in other tumors. On histopathological examination, it corresponds to epidermal damage by tumor cells and infiltration of the dermis. The presence of white circles has also been reported as a strong predictor for an invasive form of SCC [37]. On dermatoscopic examination, AK can be differentiated from the superficial forms of BCC, in which short linear vessels and pigmented structures such as radial lines converging from a common base (so-called “leaf-like areas”) or brown papules (the so-called “spoke wheel areas”) can be seen [38]. Another differential diagnosis is seborrheic keratoses, which are characterized by thick lines, good demarcation, and white clods more visible under non-polarized light (so-called “milia-like cysts”) [39]. Pigmented AKs on dermatoscopy can also be differentiated from lentigo maligna, for which asymmetrically hyperpigmented hair follicles, rhomboid structures, irregular clods, concentric circles, and poor demarcation are typical [40]. For flat lesions not yet showing typical features of this neoplasm, the so-called dermatoscopic inverse approach published by Lallas et al. is helpful [41]. It involves evaluating the dermatoscopic image to determine features of benign lesions such as scales, white and wide hair follicles, and erythema (AK), as well as features typical for seborrheic keratoses. The absence of these characteristics should give an indication for a biopsy of a doubtful lesion [41].

### 2.1. Actinic Keratosis in High Frequency Ultrasonography (HFUS)

Ultrasonography is a widely used method in medicine. It is applied in various indications, especially in the analysis of tumors and treatment response monitoring in different skin conditions, including dermatology and aesthetic medicine [42]. High-frequency devices allow visualization of the skin, appendages, and subcutaneous tissue (to the extent depending, among others, on location). Ultrasound imaging is based on the reflection of ultrasound waves by the difference in cell structure. Therefore, it might be useful in determining the margin of a hypoechogenic tumor from a hyperechogenic surrounding tissue. The higher the frequency, the better the image resolution, but the lower the penetration [43]. The most commonly used projection remains the B projection, which allows echogenicity to be determined [44]. As the main structure of the epidermis, keratin strongly reflects the ultrasound waves; thus, this layer is hyperechogenic in HFUS, and its superficial layers are responsible for the formation of entrance echo [45]. The dermis is usually hyperechogenic due to its collagen fibers [46], but its echogenicity is lower than the entrance echo. The papillary dermis may also differ from deeper parts by lower echogenicity, which is related to the arrangement of collagen fibers [47,48,49]. The description of a skin lesion should include shape, echogenicity, dimensions in all axes, and vascularization [50].

**Figure 2:**
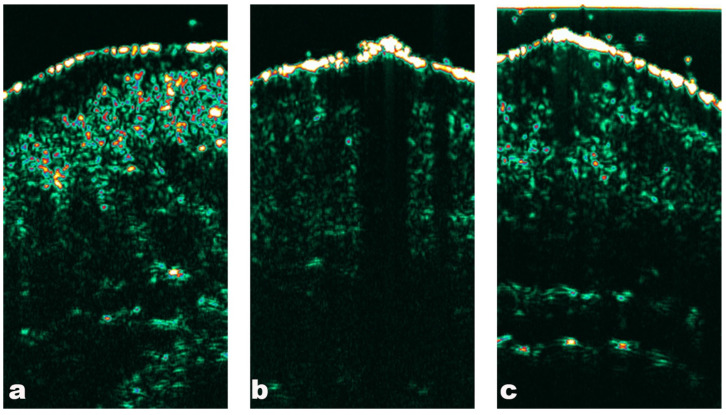
High-Frequency Ultrasonography (DermaScan C, 20 MHz) in actinic keratosis: (**a**) grade 1, (**b**) grade 2, (**c**) grade 3. Hypoechogenic structures underneath the entrance echo within the upper parts of the dermis; in higher grades, a perpendicular shadow corresponding to hyperkeratosis can be seen.

In recent years, many studies have confirmed the effectiveness of HFUS in dermato-oncology [48]. In HFUS, scales that are very prevalent in AKs are characterized by a thickened hyperechoic line or perpendicular shadow located at the epidermal level (Table 1) [47]. A study by Zhu. et al. [51], using a transducer at high frequency (22–50MHz), reported that the most common AK features amongst 54 analyzed lesions were an irregular basal border of the lesion and its regular surface. The authors underline that a single criterium in HFUS cannot be considered predictive for a certain diagnosis, while its combination with other clinical features might be useful in classifying the tumor. In their study, they used a linear array transducer operating at 10–22 MHz and an ultrasound biomicroscopy scanner with a mechanically driven linear transducer operating at 50 MHz. Analysis with a colored Doppler was also conducted. Detachment of the stratum corneum, penetrating vessels, nodular morphology, and size greater than 2 cm suggested an in situ or invasive lesion. A study by Mogesen et al. [52] on 11 AK and 23 BCC lesions reported that OCT, and especially HFUS, tended to overestimate the tumor’s thickness. Both entities were described as hypoechogenic, round-to-oval structures with a sharp border between the base of the hypoechogenic tumor and the surrounding hyperechogenic dermis (Figure 2) [51]. Arisi et al.’s [53] study on 84 patients used HFUS to monitor the treatment of AKs with methyl aminolevulinate photodynamic therapy, ingenol mebutate gel, and diclofenac gel. They assessed the epidermal and dermal thicknesses, subepidermal low-echogenic band (SLEB) thickness, and lesional and SLEB echogenicity (expressed as a percentage of dermal density) as the main HFUS features of photodamage or hyperkeratosis. In the groups of patients treated with ingenol mebutate and diclofenac gel, the measurements were similar to baseline examination, while they significantly decreased in lesions treated with methyl aminolevulinate photodynamic therapy. However, the total thickness of the dermis after treatment of AK did not change, suggesting that the therapy does not lead to skin atrophy. Methyl aminolevulinate photodynamic therapy was the only therapeutic option that significantly increased the thickness of the dermis and significantly reduced the thickness of SLEB around the treated lesions, probably secondary to neovascularization, reduction of inflammatory cell infiltration in the dermis, and removal of elastin fibers by macrophages [53].

Compared to other non-invasive diagnostic modalities, such as dermoscopy, RCM, and OCT, HFUS can overcome the limitations of illustrating the imaging depth of a lesion. Moreover, the examination is fast and cost-effective. Regardless, AK in HFUS does not have specific features, as reduced echogenicity is observed in other skin cancers. Although the diagnostic value of this method in AK is not high, and the clinical staging with HFUS is not known, its usefulness in treatment monitoring has been demonstrated. [49,50,51,52,53] It is important to mention that solar elastosis, where a typical SLEB may be observed, not associated with neoplastic infiltration, may disturb full assessment in sun-exposed areas.

### 2.2. Actinic Keratosis in Reflectance Confocal Microscopy (RCM)

RCM is a widely used noninvasive real-time skin imaging technique that allows analysis of a wide variety of skin cancers, as well as cutaneous infections and infestations [54]. Commonly available confocal microscopes use a low-power near-infrared laser beam (830-nm diode laser, power up to 35 mW) for imaging [55]. RCM imaging provides a resolution of 0.5 to 1 μm and an axial resolution of between 3 and 5 μm [56]. The laser light is a source of coherent, monochromatic light that penetrates the tissue and illuminates at a single point [57]. The cellular structures have different refraction indexes and, thus, become reflected. Then, the reflected light is captured and illustrated into a two-dimensional gray scale by the software [58]. Due to its technical capabilities, it is particularly helpful in the examination of flat lesions. Evaluation of the specimen by confocal microscopy can be performed in vivo-directly on the patient’s skin or ex vivo by analyzing surgically excised tissue. The only limitation of this method is that it enables evaluation of the lesion to a depth of 250 μm, which usually allows it to reach the papillary layer or the upper levels of the reticular layer of the skin. In RCM, the stratum corneum is usually bright due to the high refractive index of keratin, while the granular layer presents a so-called “honeycomb pattern”, which consists of keratinocytes that display polygonal, bright cellular outlines. The spinous layer might also form this kind of pattern alongside polygonal keratinocytes with refractile cytoplasm and dark central nucleus [59].

**Figure 3:**
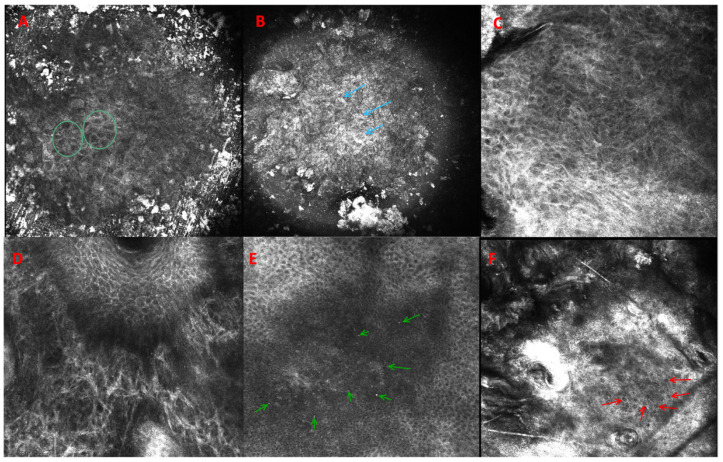
Reflectance confocal microscopy images of actinic keratosis (VivaScope 1500/3000, MAVIG, GmBH, Munich, Germany, diode laser at wavelength of 830nm). From left to right: (**A**) dark central areas of parakeratosis (circles); (**B**) detached corneocytes (arrows); (**C**) epidermal loss of honeycomb pattern; (**D**) solar elastosis; (**E**) epidermal inflammatory infiltrates (arrows); (**F**) atypical keratinocytes with nuclei of different shapes (arrows).

Over the past few years, RCM has been greatly developed, especially in the diagnosis spectrum of lentigo maligna, BCC, SCC, and AK. In a study on 48 cases of AKs, Pellacani et al. reported that the histopathological images of AKs correlated with grades of keratinocyte atypia morphology in RCM [60]. The specificity and sensitivity of RCM in diagnosis in AK depend on the study and vary between 91 and 100% and 78–100%, respectively [61]. Multiple reports have tried to assess AK features in RCM. Rishpon et al. [62] suggested that the AK features seen in RCM consisted of an adherent scale and an atypical honeycomb pattern alongside a disarranged pattern of a spinous-granular layer (Table 1). The most common vascular structures seen were the round vessels transversing dermal papilla [62]. Zalaudek et al. correlated the dermatoscopic grades of AK with their imaging in RCM [31]. In grade 1 AKs, focal areas of atypical honeycomb patterns at the level of the stratum spinosum were described, interspersed with areas of preserved, regular honeycomb patterns. In grade 2 AK, the atypia of keratinocytes is more severe, involves the stratum spinosum and granulosum layers, and is accompanied by different sizes of cells and shapes. Grade 3 AK is characterized by a markedly atypical honeycomb pattern with areas of partially disrupted epidermis, referred to as a disarranged pattern [33]. Hyperkeratotic SCC or AKs can be difficult to differentiate, as RCM does not allow imaging of lesions below the dermal-epidermal junction—only superficial components of adnexal structures are seen (Figure 3). It is a major RCM limitation, and it is sometimes difficult to differentiate AKs from SCCs during the examination. Therefore, the use of keratolytic preparations or mechanical removal of scales facilitates the evaluation of lesions suspected of malignancy. In Peppelman’s study on 30 lesions clinically suspicious for AK or SCC, the presence of architectural disarray in the stratum granulosum, in combination with architectural disarray in the spinous layer and/or tumor nest in the dermis, were the main RCM features to distinguish SCC from AK [63]. In SCC, nest-like structures and pleomorphic cells are detected in the dermis, but they are absent in AKs [64]. RCM was also useful in visualizing changes in the areas of field cancerization and subclinical AKs by presenting an atypical honeycomb pattern within the granular and spinous layers [65].

RCM has also been used as a device helpful in monitoring AK treatment. In a study conducted by Malvehy et al. [66] on a group of 14 patients, a reduction in scaling and a decrease in atypia in the honeycomb pattern were observed during the treatment of AK with a 3% diclofenac sodium in a 2.5% hyaluronic acid gel. In addition, collagen remodeling and a reduction in the inflammatory process were also observed during treatment. In Mota et al.’s [67] study on AK treatment efficacy with cryotherapy on 44 lesions, RCM was able to visualize the reduction of thickness in stratum corneum, parakeratosis, polarization of cells, keratinocyte disarrangement, and inflammation. Furthermore, studies show a reduction in keratinocyte atypia during AK treatment with imiquimod and photodynamic therapy [68,69]. Lewandrowski et al. suggested that atypical honeycomb patterns, hyperkeratosis, disarranged epidermal patterns, and stratum corneum disruptions might be the most reliable criteria for assessing AK treatment response [70]. 

Recently, Mazur et al. [71,72] suggested that RCM might also be helpful in monitoring PDT treatment response in a pigmented subtype of AK (pAK) in Caucasians. They found that the “annular granular pattern” and the presence of so-called “grayish areas” found in pAK corresponded in RCM to the presence of melanophages in the upper layer of the dermis. A number of these phenomena further increased after PDT, suggesting possible PDT-related activation of melanophages. Interestingly, RCM may be of use in assessing the doubtful pAK lesions. It can be conducted either at the beginning of the treatment when the diagnosis of the lentigo maligna must be ruled out, or at the end, when the remaining residual pigment in the lesion can be taken for unsuccessful treatment. In fact, the observed Tyndall effect can be explained by the aforementioned activated melanophages. The decision whether the lesion is fully healed (with only residual pigment) or should undergo another treatment cycle rests on RCM examination and observation of whether the lesion is still characterized by keratinocyte atypia.

### 2.3. Actinic Keratosis in Optical Coherence Tomography (OCT)

Optical coherence tomography (OCT) enables real-time imaging of skin structures in a fast and non-invasive way and allows the examination of the superficial layers of the skin in vivo in real-time. It may be applied to numerous different skin lesions, including malignancies, infectious diseases, and nail disorders [73]. It is based on interferometry, and images are captured by detecting the intensity of reflected light as a function of depth. OCT images are usually displayed in vertical and horizontal sections and can be two- or three-dimensional [74,75,76]. Compared to other non-invasive skin imaging techniques, OCT occupies a space between HFUS and RCM. In OCT, skin lesions are illustrated more accurately than in HFUS but at a lower skin level than in confocal microscopy. This method allows the skin to be evaluated at a depth of 0.4 to 2.00 mm with an optical resolution of 3 to 15 μm [75,76]. In healthy skin, the stratum corneum is visible as a thin, hyperreflective homogeneous band at the top of the image. The facial skin is very thin compared to other locations. The epidermis can be seen below the stratum corneum as a darker, homogeneous layer. It is well demarcated by the dermal-epidermal junction and the dermis, which is located at the bottom of the OCT image and represents a hyperreflective band with signal-poor areas, usually corresponding to blood or lymphatic vessels [76].

High-definition OCT (HD-OCT) is a new technique based on the same principle as standard OCT, except that its lateral and axial resolution of 3 μm allows cellular imaging of skin structures [77]. Instead of a single-pin diode light detector, it uses a two-dimensional imaging array to allow for simultaneous cross-sectional and en-face (horizontal) image acquisition [78]. The penetration depth of HD-OCT is 570 μm [79]. Therefore, this places HD-OCT in the spectrum between confocal microscopy and conventional OCT. In en-face mode, corneocytes are visible as anucleated polygonal cells, grouped into “islands” divided by skin folds. In the granular layer on HD-OCT, keratinocytes are imaged as dark areas with nucleated cells surrounded by granular and bright cytoplasm. The spinous layer presents a honeycomb pattern of smaller cells. Keratinocytes in the basal layer consist of rings of bright cells surrounding dark dermal papillae [76]. In comparison to regular OCT, HD-OCT provides a higher resolution of imaging. However, conventional OCT has a higher penetration depth than HD-OCT. Nevertheless, out of all described non-invasive imaging modalities, RCM offers the highest resolution and allows the visualization of the cellular and subcellular morphology [80]. 

For many years, studies have been conducted to determine whether OCT is applicable in NMSC. A pilot study by Korde et al. [81] involving a group of 112 patients suggested that OCT could observe signs of photodamage in the skin. They were characterized by signal enhancement in the epidermis and rapid light attenuation. The images of AK were confirmed by H&E histopathological images. Compared to RCM and HD-OCT, OCT does not allow imaging of individual cells but the assessment of individual skin layers such as the epidermis, dermo-epidermal junction, and dermal-epidermal layer. OCT can visualize typical histopathological aspects of AK, such as hyperkeratosis, epidermis thickening, and distinct boundary at a depth consistent with the epidermal-dermal junction epidermal level, as well as breakdown of cell architecture with disruption of the dermal-epidermal layer [81]. A pilot study by Barton et al. [82] on 20 AK lesions located on the forearms described a dark band in the squamous layer that had a sensitivity of about 79% and a specificity of 100% for AK. Hyperkeratosis can also affect the examination by generating artifacts as the air trapped between the scales causes a very bright OCT signal and attenuation of the deeper layer [83]. Multiple other studies confirmed typical OCT features for AK, which include the presence of a dark band (associated with epidermal thinning), hyperreflective streaks and dots, probably corresponding to dense, hyperkeratotic areas, disruptions in the epidermis and vascular reticulation [84,85,86]. 

A pilot study by Boone et al. on 17 patients with AKs attempted to correlate the different grades of KIN with their HD-OCT images (the lesions were excised right after the HD-OCT examination) [86]. They reported an atypical honeycomb pattern confined to the lower 1/3 of the epidermis, which correlated with early lesions and KIN-I. Atypical keratinocytes provoking an atypical honeycomb pattern involved mostly the lower 2/3 of the epidermis in KIN-II, while full-thickness atypia in the epidermis and chaos without honeycomb pattern was consistent with KIN-III. In all lesions, a perivascular inflammation was noticed. In addition, the authors found a correlation between morphological features in cross-sectional HD-OCT images and histopathological variants of AK. The atrophic variant with an atrophic stratum malpighi and typically a substantive overlying hyperparakeratosis was only seen in KIN-I. The typical features of hypertrophic variants were parakeratosis, acanthosis, and psoriasiform hyperplasia. In the lichenoid variant, an interface inflammatory infiltrate was detected. Lower two-thirds or full-thickness atypia was usually noticed in the Bowenoid subtype. Maier et al. [80] were able to recognize alterations of keratinocytes in AK and reported cellular and nuclear polymorphism in the en-face mode. 

Another study conducted by Boone et al. [87] attempted to identify features that would distinguish AK from SCC as well as between different AK subtypes on HD-OCT. In addition, they also reported that differentiating AK from healthy skin in HD-OCT might be possible due to disrupted epidermal architecture on cross-sectional imaging and variation in cell shape, size, and reflectivity (atypical honeycomb pattern) in one or more layers of the epidermis on en-face imaging [80]. Furthermore, the most distinctive element that helped to discriminate AK from SCC was a visualizable outline of the dermal-epidermal junction, which was present in healthy skin, as well as in most AKs, but not in SCC. In an invasive tumor, the dermal-epidermal junction is interrupted because of the presence of irregular reflective buddings projecting from the epidermis deep into the dermis or neoplastic epithelium invading around hair follicles [87,88,89]. 

Like other non-invasive imaging modalities, OCT and HD-OCT have also been used to study therapeutic efficacy in AK. Malvehy et al. [89] used HD-OCT to assess AKs and subclinical AKs treated with 0.5% fluorouracil and 10% salicylic acid. The authors examined the thickness of the stratum corneum and epidermis at the beginning of the study and two weeks after the end of treatment. HD-OCT showed a significant reduction in stratum corneum and epidermal thickness in both clinical and subclinical AK. Meanwhile, Banzhaf et al. [90] reported OCT as a valuable tool for monitoring AK therapy with imiquimod. At 3–4 weeks after finishing the treatment, disrupted layering observed in all the lesions at the baseline remained only amongst 3 out of 8 studied AKs; however, on histology, no signs of AK were detected. Furthermore, Themstrup et al. [91] suggested that OCT cannot monitor the freezing depth during cryotherapy in AKs; regardless, it was able to visualize typical AK features. In the in vivo mode, 20 min after the procedure, vesicles were located alongside the dermal-epidermal junction, separating the epidermis. Studies have also outlined OCT’s usefulness in monitoring AK treatment with ingenol mebutate [92,93]. Dermal edema and subepidermal blistering were evident on day 3 of the treatment, while slight thickening and unevenness of the epidermis were noticed after the therapy. 

#### New Perspectives

One of the recently introduced methods in the assessment of skin tumors is line-field optical coherence tomography (LC-OCT). It is a new imaging modality that is based on a combination of optical coherence tomography and reflectance confocal microscopy with line-field illumination, which presents cell-resolved images of the skin in vivo [94]. Its application in dermatooncology, including AK, has recently been published [95] and allows AKs to be differentiated from SCC [96]. Furthermore, an artificial intelligence (AI)-based PRO score assessment in AKs for LC has been published [97,98], which enhances the assessment of AKs using this noninvasive method.

Moreover, the application of HFUS in actinic keratosis remains ambiguous and may be a subject of future studies. Another promising method is super high magnification dermoscopy, which allows the magnification of 400×. However, the data on non-melanocytic skin cancer is still scarce and requires more analysis because most of the published data are focused on basal cell carcinoma [99,100].

## 3. Summary

The combination of clinical imaging and the use of non-invasive diagnostic methods increases sensitivity in diagnosing benign and malignant lesions and reduces the number of unnecessary excisions. Dermatoscopy is the most frequently used method to evaluate malignant lesions; however, OCT, RCM, and HFUS, available in some of the more specialized centers, allow for a more accurate diagnosis of difficult cases. Combining different techniques may enhance the sensitivity and specificity of the examination. The accessibility and cost-effectiveness of each device varies. Early therapy of premalignant lesions reduces the burden on dermatological offices and decreases the potential cost of treating advanced tumors, while the application of non-invasive imaging modalities makes it possible to assess the effectiveness of applied therapies. The application of non-invasive skin imaging techniques in diagnosing actinic keratosis is presented in Table 1.

**Table 1:** The overview of actinic keratosis features in non-invasive skin imaging techniques.

	Dermatoscopy	HFUS	RCM	OCT	HD-OCT
Findings	In grade one AK, a pattern of pink or red pseudonetwork with discrete white scales. Grade two: an erythematous background alongside white or yellow, keratinized and wide hair follicles (commonly known as the “strawberry pattern”). Grade three: wide hair follicles filled with keratinized masses on a scaly, white-yellow background. Other: four dots, visible only under polarized light, commonly referred to as “rosettes” (only seen in polarized mode), shiny streaks, and linear, wavy vessels surrounding the hair follicles. Pigmented variant of AK: a brown pseudonetwork and granular-annular homogeneous areas.	Round to oval structures with a sharp border between the base of the hypoechogenic tumour and the surrounding hyperechogenic dermis. Hyperkeratotic scales are characterized by thickened hyperechoic line or perpendicular shadow located in the epidermis.	In grade 1: focal areas of atypical honeycomb pattern at the level of the stratum spinosum, interspersed with areas of preserved, regular honeycomb pattern. Grade 2 AK: the atypia of keratinocytes is more severe, involves the stratum spinosum and granulosum layers, and is accompanied by different sizes of cells and shapes. Grade 3: a markedly atypical honeycomb pattern with areas of partially disrupted epidermis, referred to as a disarranged pattern.	The presence of a dark band (associated with epidermal thinning), hyperreflective streaks and dots, probably corresponding to dense, hyperkeratotic areas, disruptions in the epidermis and vascular reticulation.	An atypical honeycomb pattern confined to the lower 1/3 of the epidermis, correlates with early lesions and KIN-I. Atypical keratinocytes provoking an atypical honeycomb pattern involves mostly the lower 2/3 of the epidermis, while full thickness atypia in the epidermis and chaos without honeycomb pattern are consistent with KIN-III.
